# Healthy Ageing Should Be a Key Component of Ageing in Place: Case Study from Hong Kong

**DOI:** 10.3390/ijerph20105779

**Published:** 2023-05-10

**Authors:** Jean Woo, Rina Ko, Ruby Yu, Stacey Chan, Regina Lo, Kar Him Mo

**Affiliations:** 1 CUHK Jockey Club Institute of Ageing, The Chinese University of Hong Kong, Hong Kong SAR, China; 2 Department of Medicine & Therapeutics, Faculty of Medicine, The Chinese University of Hong Kong, Hong Kong SAR, China; 3 School of Architecture, Faculty of Social Science, The Chinese University of Hong Kong, Hong Kong SAR, China

**Keywords:** healthy ageing, ageing in place, property management

## Abstract

As part of a knowledge-transfer project consisting of a series of three talks on the topic of healthy ageing and ageing in place, we explored what participants (older adults, students, the general public, as well as professionals in architecture, urban planning and property management) consider to be key requirements for ageing in place and healthy ageing. Feedback is captured using survey questionnaires and a post-talk discussion group. Safety, a comfortable and spacious environment, age-friendly facilities and meeting the needs of older adults, the availability of caring support and home maintenance services were the most frequently mentioned desirable features of ageing in place. Future models for different types of support for ageing in place may be explored by management companies working with the residents themselves, to develop a sustainable business model.

## 1. Introduction

With the population ageing worldwide, the term ‘Ageing in Place’ has frequently appeared in the context of research as well as policies. Ageing in place is a term that covers many domains. A recent review of the existing literature identified five themes, covering place, social networks, support, technology, and personal characteristics [1]. While the concept of ageing in place is widely accepted, it may be argued that healthy ageing is an overriding concept put forward by the World Health Organization and adopted by the United Nations, who is currently promoting 2020–2030 as the UN Decade of Healthy Ageing. The latter views health as more than the absence of disease and dependence on medications, viewing health as being represented by functional ability. The latter is influenced by individual intrinsic capacity (consisting of five domains: cognitive, locomotor, vitality, sensory, and psychological), and the physical and social environment [2,3,4]. Therefore, a prerequisite of ageing in place should enable healthy ageing to take place through the provision of a physical and social environment that maximises functional ability with ageing.

Studies on ageing in place tend to emphasize the lower cost as well as the positive effect of a familiar environment on the quality of life of older people [5,6]. The majority of studies have been carried out in Western countries. Few studies have been carried out in Asian cities, which tend to be characterized by a high living density with high-rise buildings rather than individual houses. In this context, policies regarding ageing in place may differ. One study in Malaysia examined ergometric design [7], while one in Hong Kong examined the micro-, meso- and macro-environments of public-housing rental flats, concluding that most older people are satisfied with the design and supporting neighbourhood facilities [8].

A policy of ageing in place that facilitates healthy ageing in Hong Kong is particularly relevant, since it has the highest total life expectancy in the world but also has a high income inequality. However, the high total life expectancy is not accompanied by a healthy life expectancy, in that there is a rising trend of frailty and dependency [9,10]. The WHO proposes a ratio of healthy life expectancy divided by the total life expectancy at age 60 as a measure of the impact of efforts to promote healthy ageing. There are indications of a recent trend in Hong Kong of a decreasing ratio. Although the population density of Hong Kong is 6300 per square kilometre, there are many advantages in the compact urban design and high-rise housing with elevators, which is within easy reach of markets, the transport terminus, healthcare clinics, banks and various shops. The transport system is fast and efficient, neighbourhoods have a low crime rate, and the urban design includes green spaces dispersed around housing estates. Most of the housing estates in Hong Kong are managed by companies appointed by residents. These companies have management offices on site in order to deal with the running of the estate (such as building, utilities and facility maintenance, security, and, to varying extents, activity programs for residents). The built areas are in close proximity to country parks, which occupy about 50% of the total area of Hong Kong. While the physical environment may have advantages, personal factors may become more important in view of the trend in declining functional ability (or healthy ageing), and these need to be considered in the ageing-in-place discourse.

While there is no formal policy in Hong Kong for promoting ageing in place, the government has a mechanism that limits admissions to subvented residential care using a care assessment that effectively excludes those who are not dependent on others for care. Members of the public also try to avoid admission to residential care unless they cannot cope at home. Therefore, it would be pertinent to examine whether such informal ageing-in-place policies affects the healthy ageing process and whether any improvements in current living environments are needed. Since the concept of ageing in place involves many disciplines (architects, building management, urban planning, neighbourhood facilities and social support), but most importantly older people themselves, such enquiries would ideally involve people in all these categories.

As part of a knowledge-transfer project supported by the Chinese University of Hong Kong entitled ‘Healthy Aging and Ageing in Place’, we conducted a series of talks from professionals in health and social care, housing and buildings management regarding how healthy ageing may be promoted in current living environments. The audience included students of these disciplines as well as retired older adults who may or may not be caring for older family members themselves, all of whom provided opinions about what they considered to be key requirements for ageing in place and healthy ageing. This manuscript reports on the findings.

## 2. Participants and Methods

The knowledge-transfer project talks were grouped under the title of ‘Senior Health and Residential Property services’. A series of three talks were given by a geriatrician, a geriatric nurse, a fitness trainer, a dietitian, and three organizations that provide senior housing and property management services, addressing the topic of healthy ageing and ageing in place from the service providers’ perspective. They included the largest government housing authority, a non-government organization called the Housing Society, as well as a private company providing senior housing. The same list of invited participants was used for each talk. The list included older adults (mainly retired), students from architecture, social sciences, public health and medicine, as well as architects, urban planners, and staff from property management companies. Before the public talks, participants were asked to complete an online questionnaire that explored their views on age-friendly homes and the role of property management in facilitating healthy ageing. Discussions held after the talks also provided material that complemented the findings of the survey. The number of participants for each talk ranged from 71 to 78.

## 3. Results

A total of 137 people who attended the talks completed the online questionnaire. A total of 42.3% were aged 55–84, and 20.4% were aged 35–44. A large proportion (62.8%) were practitioners of architecture, property management and urban planning and design; 19.0% of participants were retired (Table 1).

Safety, a comfortable and spacious environment, age-friendly facilities and meeting the needs of older adults were the most frequent comments (Table 2). Interestingly, technological assistance was not ranked very highly. No age-related difference in ranking was observed. Of note, the following items were not mentioned by older adults themselves, but by practitioners and students: addressing the needs of older adults, empowerment, universal design, and technological assistance.

With regard to the types of services that may be provided by property management companies, there were 169 responses (Table 3). Caring support and help with home maintenance were most frequently mentioned. Caring support covered social interactions, help with first aid or alarm systems. More retirees chose home maintenance as a desirable feature, illustrating different need perspectives (Table 4).

Various themes were covered during the discussion sessions after the talks. Ninety-four percent of participants think that services provided are as important as a suitable physical environment for ageing in place. Services may contribute to functional ability where the ‘hardware’ cannot meet all needs. Additionally, the themes of psychosocial and spiritual needs, respect, and the availability of help of all kinds when needed were highlighted. Beside family support, and in particular when increasing numbers of older adults prefer not to live with their children, it was felt that property management companies have an important role to play in ageing in place. Apart from the maintenance of facilities, staff could play an important role in caring support, helping with home maintenance as well as other services such as liaising with relatives, etc. For example, one participant who lives alone mentioned that it would be very helpful to ask staff to provide advice regarding trusted, reliable handyman services for minor repairs at home. Additionally, occasional help with the moving of large objects, such as furniture, would also be very useful. Such support could be charged separately from the regular property maintenance charges. Another participant, a carer for an elderly relative who lives alone, suggested that it would be good if management staff could contact the resident on occasions when the relative expected the resident to be in but there was no answer when they phoned. There was anxiety about whether something had happened to the elderly resident. Desirable community facilities and services included fitness programs (20%); leisure and social activities (23%); health-related services (8%); and open spaces (9%).

Comments from the housing providers and property management companies showed how current practices match or differ from the above aspirations. One non-governmental organization that has been providing housing and various other community services for older adults had the philosophy that matched these aspirations the most. Their staff would have been orientated to such needs. However, they only provide a small proportion of housing for the older population. The other providers can be categorized into public and private. The former are under government administration, and management staff are mainly concerned with the safety of structures, repairs, preventing crime, etc. Although they have records of who the older adults living alone are, caring behaviour is very much left to individual staff’s characters. Some staff members are naturally more caring and socially orientated than others: for example, they will greet residents and have conversations with them. However, for some, this is not included in the job description. There was also concern that these activities are ’medical’ in nature and that they have not received formal training. For private housing, there is increasing awareness among property and management companies that are beginning to cater to older adults, designing suitable physical environments as well as working out a business model to address the needs of older adults themselves in order to enable ageing in place. There was a general perception that residents will not be prepared to pay for these extra support services. There was a general lack of understanding of how the physical and social environment can affect health and of why it is important to incorporate the health domain when creating living environments and property management services that promote healthy ageing. An underlying reason for this appears to be the fact that health is ‘medical’ and belongs to the domain of healthcare professionals. In reality, family carers of many older adults have already taken on this role, which could equally be provided by management companies on a fee-for-service basis.

## 4. Discussion

Much of the current literature in the academic discourse on ageing in place refers to Western societies [1]. Some studies focusing on active ageing highlighted the governance and challenges at the local level using Portugal as an example [11]. This paper adds to the literature by extending the enquiry to a Chinese population living in a very dense urban area in high-rise buildings, seeking input from architects, urban planners, health and social care professionals, students, and most importantly, older adults themselves. Furthermore, ageing in place is examined from the point of view of healthy ageing as promoted by the United Nations Decade of healthy ageing, with an emphasis on physical and social environments that optimise function. Previous studies tend to focus on selected domains of ageing in place [7,8]. Hong Kong is unique in that vertical living is the norm, with easy access to transport, banks, food markets, healthcare facilities, social centres, and parks, so that there is little geographic isolation. An assessment of the age-friendly city domains in Hong Kong showed that neighbourhoods are in general supportive of ageing in place [https://www.jcafc.hk/en/Resources-Centre/Publications/Baseline-Assessment-Reports.html (accessed on 7 March 2023)]. The focus of ageing in place in Hong Kong is on the home itself, the physical and social component that enables ageing at home. The observations from this study may be applicable to other high-density-living urban cities such as Singapore.

The results show that older adults’ perspective may differ from that of architects and property management companies with respect to ageing in place, emphasizing the need for citizen (or end-user) participation, particularly in the provision of senior housing, whether the initiative is in the government or private sector [12]. This study is a first attempt in defining the features of homes and housing estates that are facilitators to healthy ageing and ageing in place. Other than optimizing individuals’ functional capacity by manipulating home design, furniture, aids and technology (hardware), reducing isolation and providing help inside the home, social support, and health-promoting group activities are equally important from older adults’ perspective. In this respect, property management companies could have a very important role to play. A greater appreciation of the role of the physical and social environment in healthy ageing would be important to the continuation of ageing-in-place policies [13]. To prevent inequalities in healthy ageing in place, these concepts need to be adopted in both public as well as private sectors. Cross-sectoral collaborations including architects, urban planners, local government, property and management companies, non-governmental organizations as well as older adults themselves would be needed to develop ageing-in-place policies that incorporate healthy ageing.

There are limitations to this study. It is a preliminary pilot exploration of the topic, and the numbers are small. It is opportunistic in nature and not designed to sample the same numbers of people from different backgrounds. Nevertheless, it provides important points that could be explored either in further studies or in the design of individual housing with a focus on the needs of older adults. Future models for different types of support for ageing in place may be explored by management companies working with residents themselves, in order to develop a sustainable business model.

## 5. Conclusions

Observations from this knowledge-transfer project may be furthered explored in a more rigorous way by conducting formal quantitative and qualitative studies using stratified random sampling that includes different types of residential blocks in areas with different socioeconomic characteristics. An important point to be highlighted is the involvement of older adults in building design and property management services that facilitate ageing in place in order to maintain functional ability in the face of a declining intrinsic capacity. In general, ageing-in-place models should incorporate healthy ageing perspectives, involve older adults in a co-creation process, be developed with the participation of government and businesses, and be place-specific rather than universal and applicable across different countries and cultures. More in-depth research on this topic would guide future health and social policies in order to achieve both healthy ageing and ageing in place.

## Figures and Tables

**Table 1:** Profile of participants.

**Age Group**	**Number**	**Percentage**
19–25	10	7.3
26–34	21	15.3
35–44	28	20.4
45–54	20	14.6
55–64	39	28.5
65–74	18	13.1
75–84	1	0.7
Total	137	100
**Role**		
Practitioner	86	62.8
Retiree	38	27.8
Student	13	9.5
Total	137	100

**Table 2:** Participants’ views on features of ageing in place.

Features	Number of Responses
Safety	22
Comfortable and spacious environment	17
Age-friendly facilities	14
Addressing different needs of older people	13
Easy mobility and use of facilities	10
Enabling independence and empowerment	9
Community supports	8
Universal design	5
Interpersonal relationship	3
Technological assistance	2
Other	4
Total	107

**Table 3:** Desirable services that property management companies may provide.

Types of Elderly Services	Number of Responses
Caring support	39
Home maintenance	20
Age-friendly facilities	12
Customised services	12
Recreation and leisure	12
Communication	9
Healthcare facilities and services	9
Social activities	9
Catering	5
Cleaning services	5
Information dissemination	5
Counselling	4
Fitness activities and facilities	4
Safe living environment	4
Shopping	4
Travelling assistance	4
Other	12
Total	169

**Table 4:** Opinions on service provision from property management stratified by roles.

Elderly Services	Roles							
	Practitioner (86)		Retiree (38)		Student (13)		Total (137)	
	N	%	N	%	N	%	N	%
Age-friendly facilities	8	9.30	2	5.26	2	15.38	12	8.76
Caring support	25	29.07	10	26.32	4	30.77	39	28.47
Catering	2	2.33	3	7.89	0	-	5	3.65
Cleaning services	1	1.16	2	5.26	2	15.38	5	3.65
Communication	6	6.98	3	7.89	0	-	9	6.57
Counselling	4	4.65	0	-	0	-	4	2.92
Customized services	8	9.30	4	10.53	0	-	12	8.76
Fitness activities and facilities	3	3.49	1	2.63	0	-	4	2.92
Healthcare facilities and services	8	9.30	1	2.63	0	-	9	6.57
Home maintenance	10	11.63	10	26.32	0	-	20	14.60
Information dissemination	1	1.16	4	10.53	0	-	5	3.65
Other	6	6.98	4	10.53	2	15.38	12	8.76
Recreation and leisure	6	6.98	4	10.53	2	15.38	12	8.76
Safe living environment	3	3.49	1	2.63	0	-	4	2.92
Shopping	1	1.16	3	7.89	0	-	4	2.92
Social activities	6	6.98	3	7.89	0	-	9	6.57
Travelling assistance	2	2.33	0	-	2	15.38	4	2.92
Total	100		55		14		169	

## Data Availability

The data presented in this study are available on request from the corresponding author.
